# Rare coding mutations identified by sequencing of Alzheimer disease genome‐wide association studies loci

**DOI:** 10.1002/ana.24466

**Published:** 2015-07-28

**Authors:** Badri N. Vardarajan, Mahdi Ghani, Amanda Kahn, Stephanie Sheikh, Christine Sato, Sandra Barral, Joseph H. Lee, Rong Cheng, Christiane Reitz, Rafael Lantigua, Dolly Reyes‐Dumeyer, Martin Medrano, Ivonne Z. Jimenez‐Velazquez, Ekaterina Rogaeva, Peter St George‐Hyslop, Richard Mayeux

**Affiliations:** ^1^ Taub Institute for Research on Alzheimer's Disease and the Aging Brain, Columbia University Medical CenterNew YorkNY; ^2^ Gertrude H. Sergievsky CenterCollege of Physicians and Surgeons, Columbia UniversityNew YorkNY; ^3^ Departments of Neurology, College of Physicians and Surgeons, Columbia UniversityNew YorkNY; ^4^ Systems BiologyColumbia UniversityNew YorkNY; ^5^ Tanz Centre for Research in Neurodegenerative Diseases and Department of MedicineUniversity of TorontoTorontoOntarioCanada; ^6^ Department of EpidemiologyMailman School of Public Health, Columbia UniversityNew YorkNY; ^7^ Department of MedicineCollege of Physicians and Surgeons, Columbia UniversityNew YorkNY; ^8^ School of Medicine, Mother and Teacher Pontifical Catholic UniversitySantiagoDominican Republic; ^9^ Department of MedicineGeriatrics Program, School of Medicine, University of Puerto RicoSan JuanPuerto Rico; ^10^ Cambridge Institute for Medical ResearchDepartment of Clinical NeurosciencesUniversity of CambridgeCambridgeUnited Kingdom; ^11^ Department of PsychiatryCollege of Physicians and Surgeons, Columbia UniversityNew YorkNY

## Abstract

**Objective:**

To detect rare coding variants underlying loci detected by genome‐wide association studies (GWAS) of late onset Alzheimer disease (LOAD).

**Methods:**

We conducted targeted sequencing of *ABCA7, BIN1, CD2AP, CLU, CR1, EPHA1, MS4A4A/MS4A6A,* and *PICALM* in 3 independent LOAD cohorts: 176 patients from 124 Caribbean Hispanics families, 120 patients and 33 unaffected individuals from the 129 National Institute on Aging LOAD Family Study; and 263 unrelated Canadian individuals of European ancestry (210 sporadic patients and 53 controls). Rare coding variants found in at least 2 data sets were genotyped in independent groups of ancestry‐matched controls. Additionally, the Exome Aggregation Consortium was used as a reference data set for population‐based allele frequencies.

**Results:**

Overall we detected a statistically significant 3.1‐fold enrichment of the nonsynonymous mutations in the Caucasian LOAD cases compared with controls (*p* = 0.002) and no difference in synonymous variants. A stop‐gain mutation in *ABCA7* (E1679X) and missense mutation in *CD2AP* (K633R) were highly significant in Caucasian LOAD cases, and mutations in *EPHA1* (P460L) and *BIN1* (K358R) were significant in Caribbean Hispanic families with LOAD. The *EPHA1* variant segregated completely in an extended Caribbean Hispanic family and was also nominally significant in the Caucasians. Additionally, *BIN1* (K358R) segregated in 2 of the 6 Caribbean Hispanic families where the mutations were discovered.

**Interpretation:**

Targeted sequencing of confirmed GWAS loci revealed an excess burden of deleterious coding mutations in LOAD, with the greatest burden observed in *ABCA7* and *BIN1*. Identifying coding variants in LOAD will facilitate the creation of tractable models for investigation of disease‐related mechanisms and potential therapies. Ann Neurol 2015;78:487–498

The first large‐scale genome‐wide association studies (GWAS) using common single nucleotide polymorphisms (SNPs) identified *CLU*, *PICALM*, *CR1,* and *BIN1* as late onset Alzheimer disease (LOAD) susceptibility loci,^1^, ^2^, ^3^ which were widely confirmed by others.^4^, ^5^ The effect sizes of these genetic associations were much smaller than for *APOE*,^2^, ^6^ with odds ratios ranging from 1.16 to 1.20. Follow‐up GWAS identified additional LOAD susceptibility variants.^7^, ^8^ Although the known function of the genes implicated in these GWAS encode proteins implicating disruptions of lipid metabolism, immune response, and endocytosis or intracellular trafficking as potential mechanisms in LOAD, only a handful of disease‐associated variants in these genes, such as *SORL1*,^9^, ^10^ have been identified.

Surprisingly, targeted exome sequencing of large multiplex pedigrees with LOAD identified mutations in *APP, PSEN1*, and *PSEN2*,^11^, ^12^ indicating that rare coding sequence variants even in genes associated with early onset Alzheimer disease (AD) may account for a portion of disease risk in LOAD. Also, rare coding sequence variants in *ADAM10*,^13^ *TREM2*,^12^, ^14^ and *PLD3* ^15^ have been found in patients with LOAD. Because the majority of loci detected by SNP‐based GWAS of LOAD have not been investigated for rare coding sequence variants, we conducted targeted sequencing of the top 8 genetic loci frequently associated with LOAD,^7^, ^8^, ^16^, ^17^, ^18^ with the exception of the *CD33* locus, which was not well replicated in subsequent large meta‐GWAS.^18^

## Subjects and Methods

### Sample Selection

All participants (Table 1) were recruited after providing informed consent and with approval by the relevant institutional review boards. Persons deemed unaffected were required to have had documented cognitive testing and clinical examination to verify their clinical status and diagnosis. Families in which patients had known mutations in *APP*, *PSEN1*, *PSEN2*, *GRN*, or *MAPT* were excluded. All selected probands came from families with ≥4 affected individuals.

**Table 1:** Demographics of the Samples in the Targeted Sequencing Experiment

	Status	No.	Mean Age at Onset or Last Examination, yr ± SD	Women, No. (%)	*APOE* ε4, %
Targeted sequencing					
NIA‐LOAD	Affected	120	75.1 ± 8.3	77 (64.2)	13.6
	Unaffected	33	82.2 ± 10.8	24 (72.7)	34.1
Toronto	Affected	210	73.9 ± 7.3	106 (50.4)	35.7
	Unaffected	53	80.3 ± 3.6	33 (62.3)	22.4
Hispanics	Affected	176	74.8 ± 8.3	111 (63.1)	25.3
Follow‐up genotyping					
Hispanics	Unaffected	444	81.2 ± 7.1	302 (68.0)	12.9
WHICAP	Unaffected	300	84.7 ± 5.5	174 (58.0)	10.0
Toronto	Unaffected	238	73.1 ± 9.4	137 (57.5)	14.4

LOAD = late onset Alzheimer disease; NIA = National Institute on Aging; SD = standard deviation; WHICAP = Washington Heights‐Inwood Community Aging Project.

### National Institute on Aging–LOAD/National Cell Repository for Alzheimer's Disease Study

Affected and unaffected individuals (n = 153) from 129 families within the National Institute on Aging (NIA)‐LOAD Family Study^5^ were selected for targeted sequencing analysis, including 120 individuals with LOAD and 33 similarly aged unaffected subjects (see Table 1). Patients had a mean age of onset of 75.1 ± 8.3 years with 38% frequency of the *APOE* ε4 allele, and unaffected participants were older (mean age = 82.2 ± 10.8 years) with 13% frequency of the *APOE* ε4 allele.

### Family Study of Genetic Influence in AD

Recruitment for the Family Study of Genetic Influence in AD (Estudio Familiar de Influencia Genetica en Alzheimer) began in 1998, and was restricted to Caribbean Hispanics,^19^ mostly from the Dominican Republic. A total of 176 affected patients from 124 families were selected for targeted sequencing (the mean age of onset was 74.8 ± 8.3 years; 63.1% were woman).

### Toronto LOAD Study

Targeted sequencing of GWAS loci was conducted in 210 well‐characterized sporadic LOAD patients and 53 normal controls of European ancestry from the GenADA study based on sufficient quantity/quality DNA samples. The mean age of onset in cases was 73.9 ± 7.3 years, 50.4% were women, and *APOE* ε4 allele frequency was 35.7%. These patients were either clinically diagnosed with probable LOAD (n = 169) or autopsy‐confirmed LOAD cases from the brain bank at the Tanz Centre for Neurodegenerative Research in Toronto (n = 41). Mean age at the time of examination in controls was 80.3 ± 3.6 years, 62.3% of them were women, and the *APOE* ε4 allele frequency was 22.4%.

### Exome Aggregation Consortium

The Exome Aggregation Consortium (ExAC; http://exac.broadinstitute.org) data set was used as a reference data set of population‐based allele frequencies. It contains data from 60,706 unrelated adult individuals sequenced as part of various disease‐specific and population genetic studies from 6 different ethnic groups. LOAD was not among the diseases investigated. We used the non‐Finnish European and Latino/American cohorts to compare the frequencies of variants found in Caucasian and Caribbean Hispanic cohorts, respectively.

### Sample Preparation

High molecular weight DNA was isolated from either fresh or frozen samples that had been stored at −80^o^C. Blood genomic DNA was isolated using the Gentra Puregene and FlexiGene kits (Qiagen, Valencia, CA), and saliva genomic DNA was isolated using prepIT.L2P (DNA Genotek, Kanata, Ontario, Canada). When high‐quality DNA derived from blood was unavailable, lymphocyte cell lines were used (in a total of 13 probands). DNA concentration was determined by NanoDrop (Thermo Fisher Scientific, Waltham, MA) for most analyses.

### Targeted Sequencing

Target enrichment of the samples was performed using the Agilent Technologies (Santa Clara, CA) SureSelect system (for Hispanics and the NIA‐LOAD data set), and Roche (Basel, Switzerland) NimbleGen SeqCap EZ Designs‐custom (for the Toronto data set). Custom oligonucleotide baits captured exonic regions and splice sites of the genes of interest and amplified them. For the SeqCap EZ approach, the sequencing library was hybridized to the SeqCap EZ Oligo pool that was made against the target regions of interest. The end product was subjected to high throughput sequencing. After the DNA samples were prepared, they were multiplexed with index “barcode” primers and pooled for sequencing in batches of up to 12 samples.

Sequencing of all samples occurred on Illumina's Genome Analyzer IIx, HiSeq 2000, and MiSeq platforms (http://www.illumina.com). Paired‐end reads were performed over 82 to 307 sequencing cycles. Data files were demultiplexed by barcode to separate pooled samples into individual probands. We were able to obtain high coverage at an average depth of >1,000× per sample and interval region captured.

### Follow‐up Genotyping

Caribbean Hispanics with mutations also observed in 1 of the other 2 data sets underwent genotyping or Sanger sequencing to confirm nonsynonymous variants. To determine the population frequencies for variants discovered within our data sets, we genotyped unrelated controls of the same ethnic background (see Table 1). We also conducted validation genotyping in 13 Caribbean Hispanic families (n = 148) of the patients where the variants were discovered. Additionally, to compare the allele frequencies for novel variants identified in this study from unaffected persons in the Caribbean Hispanic population, we genotyped 444 unaffected and unrelated persons (68.0% women, mean age at examination = 81.2 ± 7.1, *APOE* ε4 allele frequency = 12.9%) of the same ancestry. We also genotyped 300 white, non‐Hispanic controls (58.0% women, mean age at time of examination = 84.7 ± 5.5 years, *APOE* ε4 allele frequency = 10.0%) in the NIA‐LOAD data set to estimate population frequencies for the mutations discovered. The controls were determined to be of the same ethnic background as the familial cases using methods described previously.^19^ Follow‐up genotyping was also done on 238 normal controls matched to the Toronto sporadic LOAD data set by ethnic origin, sex, and age (57.5% women, mean age at time of examination = 73.1 ± 9.4 years, *APOE* ε4 allele frequency = 14.4%). Genotypes were generated using MassArray iPLEX technology (Sequenom, San Diego, CA), following the manufacturers’ instructions. The system involves multiplex polymerase chain reaction and minisequencing assays, followed by matrix‐assisted laser desorption/ionization time of flight mass spectrometry analysis.

### Analytical Methods

We aligned the reads obtained from the pooled sequencing to the human reference genome build 37 using the Burrows–Wheeler Aligner^20^ (http://bio‐bwa.sourceforge.net). Quality control of the sequencing data was done using established methods, including base alignment quality calibration and refinement of local alignment around putative indels using the Genome Analysis Toolkit (GATK).^21^ Variants were called and recalibrated using multisample calling with GATK's UnifiedGenotyper and VariantRecalibrator modules. Reliably called variants were annotated by ANNOVAR^22^ including in silico functional prediction using POLYPHEN^23^ and extent of cross‐species conservation using PHYLOP.^24^

### Burden Tests

We estimated the burden of different classes of mutations (loss of function, all nonsynonymous, and all synonymous variants) using a binomial test as described in Neale et al.^25^ To determine whether a class of mutations was enriched in cases, we used a binomial test with probability of success equal to the frequency of mutation class in controls (background or expected frequency). Also, any bias introduced in the test due to an unbalanced case–control set was compared to observations from the synonymous mutation class that was used to set the background expectation.

### Individual Single Nucleotide Variant Significance Tests

To test the association of individual single nucleotide variants (SNVs) with LOAD, we compared the allele frequencies of SNVs in patients with unaffected samples from follow‐up genotyping combined with the publicly available ExAC data using Fisher exact test. We used this data set to provide a much larger and more representative estimate of allele frequencies than could be ascertained from the smaller NIA‐LOAD and Toronto data sets alone. Because of the lack of an optimal ethnically matched control data set for Caribbean Hispanics, we used the Latino American cohort for an estimate of allele frequencies of rare variants. Additionally, for the Caribbean Hispanic cohort only, we tested segregation and LOAD association in this data set using generalized estimation equations to adjust for familial correlation.

## Results

### Sequencing

We identified 12 coding mutations in 7 genes in at least 2 of the 3 data sets, including 7 autopsy‐confirmed LOAD cases (Table 2). These 12 coding mutations included: 4 mutations in *ABCA7*, 2 each in *CD2AP* and *PICALM*, and 1 each in *BIN1, CLU, EPHA1*, and *MS4A6A*. Three rare coding mutations were observed in cases from all 3 data sets: rs138047593 in *BIN1*, rs202178565 in *EPHA1*, and rs138650483 in *MS4A6A*; the *EPHA1* and *BIN1* mutations were subsequently confirmed by follow‐up genotyping in the Hispanic and Caucasian cohorts. The *EPHA1* variant was absent in the genotyped Caucasian controls and the *BIN1* variant had the same frequency as the Caucasian LOAD cases. Thus, we assessed the association of these variants independently in Caucasian and Hispanic cohorts by comparing them against the population‐based allele frequencies available in the ExAC database and by testing family‐based association in Caribbean Hispanic families.

**Table 2:** Annotation of Rare or Novel Nonsynonymous Single Nucleotide Polymorphisms Found in at Least 2 of the 3 Data Sets

Chr	Position	ID	Ref	Alt	Function	Gene	AA Change	POLYPHEN	SIFT
2	127808046	rs138047593^a^	T	C	Nonsynonymous	*BIN1*	K358R	D	D
6	47563608	rs138727736	A	G	Nonsynonymous	*CD2AP*	T374A	B	B
6	47591941	rs116754410	A	G	Nonsynonymous	*CD2AP*	K633R	D	D
7	143095499	rs202178565^b^	G	A	Nonsynonymous	*EPHA1*	P460L	D	P
8	27462662	rs41276297	G	A	Nonsynonymous	*CLU*	T203I	B	B
11	59940500	rs138650483^a^	C	T	Exonic/splicing	*MS4A6A*	V218M	D	D
11	85687719	rs147556602	G	C	Nonsynonymous	*PICALM*	P495A	D	D
11	85701307	rs117411388	T	C	Nonsynonymous	*PICALM*	H458R	D	D
19	1041971	rs201665195	T	G	Nonsynonymous	*ABCA7*	L101R	D	D
19	1051006	rs143718918	G	A	Nonsynonymous	*ABCA7*	R880Q	D	D
19	1057343	rs117187003	G	A	Nonsynonymous	*ABCA7*	V1599M	D	D
19	1058154	novel	G	T	Stop‐gain	*ABCA7*	E1679X	—	—

a Found in all 3 data sets.

b Found in all 3 data sets, not found in any unaffected subjects in targeted sequencing.

### Caucasians

For the 12 variants detected in the NIA‐LOAD and Toronto data sets (Table 3), we compared the frequency of SNVs between 330 cases in these data sets and the 33,370 non‐Finnish Europeans from ExAC using a Fisher exact test. A stop‐gain mutation in *ABCA7* (E1769X) and missense mutation in *CD2AP* (K633R) were highly significant after correction for multiple testing (*p* = 5.3E‐04 and 5.3E‐08, respectively). Of the remaining variants discovered in multiple data sets, 1 rare variant in both *EPHA1* and *PICALM* was nominally significant (*p* = 0.03 and 0.007, respectively). The p.K358R variant in *BIN1* was observed in 1.8% of the ExAC database Caucasians, which is similar to the frequency we observed in the cohort of patients here with LOAD. The remaining variants were extremely rare (minor allele frequency [MAF] < 0.5%) in the ExAC database Caucasians.

**Table 3:** Allele Frequency and Fisher Tests of Single Nucleotide Polymorphisms in Caucasians

		Targeted Sequencing, Carriers				Targeted Sequencing Frequency			
Gene	ID	NIA‐LOAD		Toronto (autopsy cases)		NIA‐LOAD	Toronto		
		Unaffected	LOAD	Unaffected	LOAD	LOAD	LOAD	ExAC Frequency in Europeans	*p*, Fisher Test
*BIN1*	rs138047593	0	1	0	7 (4)	0.004386	0.016667	1.82E‐02	3.69E‐01
*CD2AP*	rs138727736	0	0	0	4 (2)	0	0.009524	4.73E‐03	1.37E‐01
*CD2AP* a	rs116754410	0	0	0	1 (1)	0	0.002381	3.06E‐05	5.33E‐08
*EPHA1* a	rs202178565	0	1	0	1	0.004386	0.002381	4.05E‐04	3.07E‐02
*CLU*	rs41276297	0	0	0	2	0	0.004762	2.51E‐03	2.82E‐01
*MS4A6A*	rs138650483	1	1	0	1	0.00431	0.002381	3.76E‐03	1.00E+00
*PICALM*	rs147556602	0	0	1	0	0	0	3.61E‐04	1.00E+00
*PICALM* a	rs117411388	0	2	0	2	0.00885	0.004762	1.11E‐03	6.84E‐03
*ABCA7*	rs201665195	0	1	0	1	0.004348	0.002381	1.14E‐03	1.82E‐01
*ABCA7*	rs143718918	0	1	0	1	0.004425	0.002381	2.11E‐03	3.89E‐01
*ABCA7*	rs117187003	0	4	1	1	0.017857	0.002381	4.16E‐03	2.01E‐01
*ABCA7* a	19:1058154	0	1	0	1	0.004425	0.002381	3.02E‐05	5.34E‐04

a Nominally significant single nucleotide variants.

ExAC = Exome Aggregation Consortium; LOAD = late onset Alzheimer disease; NIA = National Institute on Aging.

### Caribbean Hispanics

**Figure 1:**
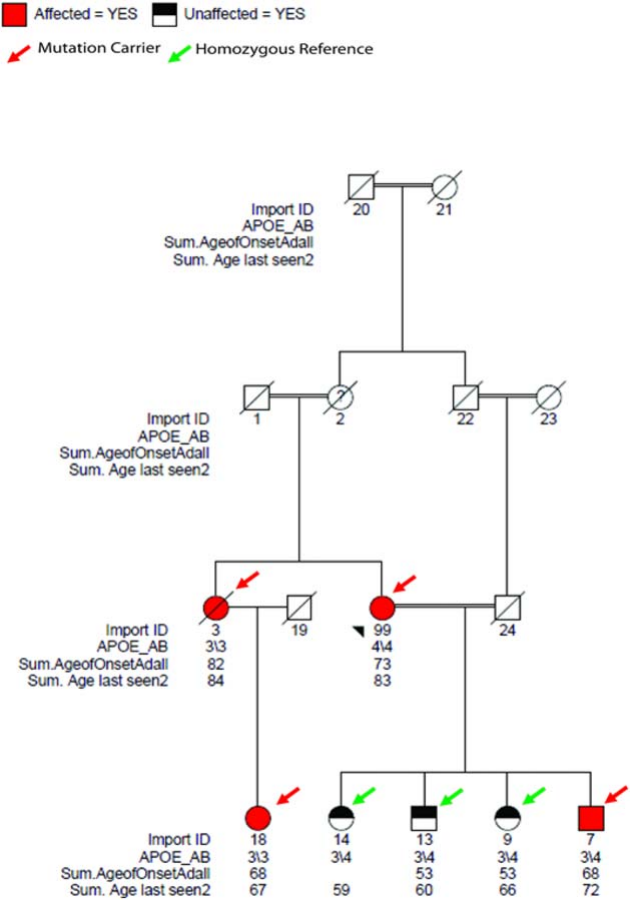
Missense damaging mutation rs202178565 in *EPHA1* (ephrin type‐A receptor 1). This mutation was not found in any external controls. Import ID = internal subject ID; APOE_AB = *APOE* ε4 status; Sum.AgeofOnsetAdAll = age at onset of disease; Sum.Age last seen2 = age of the last examination of the subject.

For the 7 variants found in this data set (and at least 1 other Caucasian data set; Table 4), we tested segregation and LOAD association using validation‐genotyping data in 13 families and 444 independent case–controls. Furthermore, we compared the frequency of the variants in LOAD patients with the Latino allele frequencies (n = 5,789) in the ExAC database. The p.P460L in *EPHA1* and p.K358R in *BIN1* were significantly associated with LOAD when compared to both internally genotyped Caribbean Hispanic controls and population Latino controls in the ExaC database after correction for multiple testing (Table 4). Notably, the *EPHA1* rs202178565 variant (P460L) was observed in only 1 of the 444 unaffected Caribbean Hispanic individuals and none of the Caucasian controls. This *EPHA1* mutation also segregated completely in 4 affected members of a Caribbean Hispanic family (Fig 1). The variant was significant both in the Fisher exact test (*p* = 2.6E‐03) and regression model (*p* = 8.64E‐05) in Caribbean Hispanics and nominally significant in the Caucasian cohort (*p* = 0.03).

**Table 4:** Allele Frequency and Association Tests in Hispanics

Gene	ID	Targeted Sequencing, Affected Carriers	Targeted Sequencing, Affected Carriers, Frequency	Control Frequency	Familial Case Frequency	Familial Control Frequency	Beta	p	ExAC Frequency in Latinos	p, Fisher Test
*BIN1* a	rs138047593	6	0.01705	0.0084	0.0859	0.0641	2.03	1.27E‐05	2.60E‐03	5.85E‐04
*CD2AP*	rs138727736	2	0.00568	0.0108	0.0238	0.0128	1.26	3.04E‐02	3.78E‐03	3.91E‐01
*CD2AP*	rs116754410	2	0.00568	0.0160	0.0323	0.0128	0.69	3.37E‐01	1.77E‐03	1.41E‐01
*EPHA1* a	rs202178565	2	0.00568	0.0011	0.0078	0	3.44	1.25E‐04	8.64E‐05	2.55E‐03
*CLU*	rs41276297	1	0.00284	0.0012	0	0			1.04E‐03	3.23E‐01
*MS4A6A*	rs138650483	1	0.00284	0.0049	0.0085	0	0.63	4.56E‐01	4.16E‐03	1.00E+00
*PICALM*	rs147556602	1	0.00472	0.0037	0.0154	0	1.21	1.81E‐01	7.84E‐04	1.67E‐01

a Nominally significant single nucleotide variants.

ExAC = Exome Aggregation Consortium.

Follow‐up genotyping of the *BIN1* p.K358R mutation revealed that it was predominantly found in affected members with LOAD from 6 Caribbean Hispanic families. We observed *BIN1* p.K358R in 11 LOAD patients and only 3 elderly controls (>65 years) in these families. We also observed the mutation in 9 unaffected members younger than 65 years (average age = 54 years). We observed a higher frequency of the mutation in the families (0.085 in familial cases and 0.069 in familial controls) compared to genotyped Caribbean Hispanic controls (0.0084) and Latino population controls from the ExAC database (0.0026). This variant was significantly associated with Caribbean Hispanic LOAD families in both a regression model (*p* = 1.27E‐05) and by Fisher exact test (*p* = 5.85E‐04). The *BIN1* p.K358R allele frequencies in Caucasian and Caribbean Hispanic population controls were similar. However, we found much higher frequency of this variant in families, suggesting that the effect of this variant in multiplex families may be due to epistasis with other genetic or environmental risk factors. Further investigation of this mutation is required to evaluate the effect of this variant in LOAD pathogenesis.

### Other Mutations

In addition to mutations observed in multiple data sets, a total of 88 rare damaging mutations were found to be present in individual data sets and were only detected in patients with LOAD: 21 in NIA‐LOAD, 37 in the Toronto data set, and 30 in the Caribbean Hispanics. When compared to the ExAC population frequencies, 38 of 88 variants were nominally significant at *p* < 0.05 (Supplementary Table), 21 of which were observed in *ABCA7* and 5 in *EPHA1*. All the nominally significant variants were extremely rare in the general population (max MAF = 0.05%), and a majority of them were predicted to be deleterious to the coding protein.

### Burden Tests

We calculated the overall burden of these novel or rare coding nonsynonymous mutations (including SNVs and short indels) compared with the burden of synonymous mutations in cases and controls for each gene in the 3 data sets (Table 5). Combining the observations from the NIA‐LOAD and Toronto Caucasian data sets, we detected a statistically significant 3.1‐fold enrichment of the nonsynonymous mutations in cases versus controls (*p* = 0.002). The LOAD cases also carried 2.76 times more loss of function mutations (stop‐loss, stop‐gain, frameshift, or splicing) and damaging missense mutations compared to controls (*p* = 0.02). In contrast, we did not observe a difference in synonymous mutations for LOAD cases in the 2 Caucasian data sets compared with controls (1.07‐fold, *p* = 0.59). The mutation rate per Caribbean Hispanic LOAD patient was comparable to that in the Caucasian data set across all genes (see Table 5).

**Table 5:** Number of Mutations (Mutation Rate per Subject) in the 3 Different Mutation Classes for Each Gene in the NIA‐LOAD, Toronto, and Hispanic Data Sets

	Cases			Controls		
Data Set	C1	C2	C3	C1	C2	C3
NIA‐LOAD data set						
*ABCA7*	12 (0.100)	15 (0.125)	2 (0.0170)	0	0	0
*BIN1*	1 (0.008)	2 (0.017)	3 (0.025)	0	0	2
*CD2AP*	2 (0.017)	2 (0.017)	2 (0.017)	1	1	1
*CLU*	1 (0.008)	1 (0.008)	1 (0.008)	0	0	0
*CR1*	0	1 (0.008)	2 (0.017)	0	0	0
*EPHA1*	3 (0.025)	3 (0.025)	2 (0.017)	0	0	0
*MS4A6A*	1 (0.008)	1 (0.008)	0	1	1	0
*PICALM*	0	3 (0.025)	0	0	0	0
Total	20	28	12	2	2	3
Toronto data set						
*ABCA7*	16 (0.075)	16 (0.075)	5 (0.023)	1	1	0
*BIN1*	8 (0.038)	8 (0.038)	4 (0.019)	0	0	4
*CD2AP*	4 (0.019)	8 (0.038)	3 (0.014)	1	2	0
*CLU*	0	2 (0.009)	5 (0.023)	0	0	1
*CR1*	1 (0.005)	1 (0.005)	9 (0.042)	0	0	1
*EPHA1*	1 (0.005)	5 (0.023)	6 (0.028)	0	0	2
*MS4A6A*	1 (0.005)	2 (0.009)	0	0	0	0
*PICALM*	2 (0.009)	2 (0.009)	1 (0.005)	1	1	0
Total	33	44	33	3	4	8
Burden test						
No.	53	72	45	5	6	11
Enrichment (*p*)	2.76 (0.02)	3.1 (0.002)	1.07 (0.59)			
Hispanic data set						
*ABCA7*	15 (0.085)	17 (0.097)	12 (0.068)			
*BIN1*	2 (0.011)	9 (0.051)	8 (0.045)			
*CD2AP*	0	4 (0.023)	0			
*CLU*	1 (0.006)	4 (0.023)	5 (0.028)			
*CR1*	0	4 (0.023)	2 (0.011)			
*EPHA1*	8 (0.045)	8 (0.045)	6 (0.034)			
*MS4A6A*	0	1 (0.006)	0			
*PICALM*	0	1 (0.006)	0			
Total	26	48	33			

C1 = class I: loss of function (stop‐gain, stop‐loss) and damaging missense mutations; C2 = class II: all nonsynonymous mutations; C3 = class III: all synonymous mutations; LOAD = late onset Alzheimer disease; NIA = National Institute on Aging.

In total, 11.1% of all patients with LOAD from 3 data sets were carriers of at least 1 coding *ABCA7* mutation. Remarkably, 47% of all potentially damaging mutations were observed in the *ABCA7* gene. Of the rare mutations, 8% were detected in *EPHA1*, affecting 3.1% of investigated LOAD cases and only a single Caribbean Hispanic control. These results are striking because based on a recent study^26^ of thousands of exomes, *ABCA7* and *EPHA1* are highly conserved genes and ranked in the top 2nd and 11th percentiles, respectively, for intolerance toward mutation in the general population. The high mutation rate in LOAD compared to controls in the highly conserved *ABCA7* and *EPHA1* implies a putative functional role in the pathogenesis of LOAD.

*BIN1* was a strong contributor to the increased mutation rate in cases compared to controls, showing damaging variants in 19 cases (3.75%) but none in controls (see Table 2 and Supplementary Table). The most frequent mutation in the patients was in *BIN1* (p.K358R), where we identified carriers in 8 Caucasian (including 4 autopsy cases) and 6 Hispanic patients.

There is prior evidence of increased expression of *ABCA7*, *BIN1*, and *MS4A6A* in LOAD brains,^27^ and increased *ABCA7* expression is associated with clinical dementia rating,^28^ with higher expression being associated with more advanced cognitive decline. *BIN1* expression levels were associated with disease progression, where higher expression was associated with a delayed age at onset. However, there was no evidence of differential expression of *EPHA1* in LOAD compared with controls.^28^

## Discussion

The results presented here imply that the loci from GWAS associated with LOAD likely contain multiple rare, damaging mutations that can be recurrent among unrelated patients and in some instances can segregate within families. The dense coverage we used for targeted sequencing allowed for the identification of variants that might not have been detectable with the more sparse coverage used in current whole exome or whole genome approaches. Variants in *BIN1* (p.K358R), *EPHA1* (p.P460L) were found in patients with LOAD from all 3 data sets and were statistically significant in follow‐up genotyping in the Caribbean Hispanic dataset. In the 2 Caucasian data sets, we found statistically significant variants in *CD2AP* and *ABCA7*. The nominally significant variants from individual data sets (see Supplementary Table) were observed in the ExAC data set at very low frequencies, providing further support that greater depth of targeted sequencing allows identification of very rare events.

In 3 data sets enriched by families multiply affected with LOAD, we sequenced 8 GWAS loci with consistent SNP‐based associations with LOAD across multiple investigations.^18^ Analysis of 2 Caucasian data sets revealed a significantly greater burden of rare and novel nonsynonymous (including SNVs and indels) alterations (*p* = 0.002) in cases compared to controls, whereas the mutation rate of synonymous variants was the similar in cases and controls. In LOAD we also observed a significant (*p* = 0.02) 3‐fold enrichment in the subset of alterations that were predicted to be damaging (by POLYPHEN or SIFT).

The greatest burden of damaging sequence variants was found in *ABCA7*. Among Caucasian LOAD cases, we detected 38 carriers of rare variants (20 in NIA‐LOAD and 18 in the Toronto data set), constituting 11.8% of 330 investigated cases, whereas only 1 carrier of such a variant was found among the 86 sequenced controls (1.2%; see Table 2 and Supplementary Table). In addition to nonsynonymous *ABCA7* variants, we observed a splice site, a stop mutation, and frameshift deletions, suggesting a loss‐of‐function mechanism associated with LOAD. Our recent functional studies of ABCA7 strongly support such a possibility,^29^, ^30^ because suppression of ABCA7 in vitro and in vivo resulted in an elevation of amyloid production. The complex function of ABCA7 includes mediation of the biogenesis of high‐density lipoprotein with cellular lipid and helical apolipoproteins,^31^ as well as function in apolipoprotein‐mediated phospholipid and cholesterol efflux from cells.^32^ Finally, a direct role of ABCA7 in APP processing may be associated with its primary biological function to regulate endocytic pathways.^30^ Importantly, we previously identified *ABCA7* as a major genetic risk LOAD locus in African Americans,^33^ and a whole‐genome sequencing study in a large Icelandic cohort identified excess burden of rare loss of function variants in *ABCA7* in LOAD.^34^ We confirmed 2 *ABCA7* loss of function variants reported in that study (c.4416+2T>G and p.Leu1403Argfs*7) and discovered 3 additional variants (p.708_710del, p.R1489X, and E1679X). Our analyses confirm that *ABCA7* has the highest burden of deleterious variants in LOAD, but differences in the observed mutations could be due to ethnicity, capture, and coverage differences in the 2 studies.

**Figure 2:**
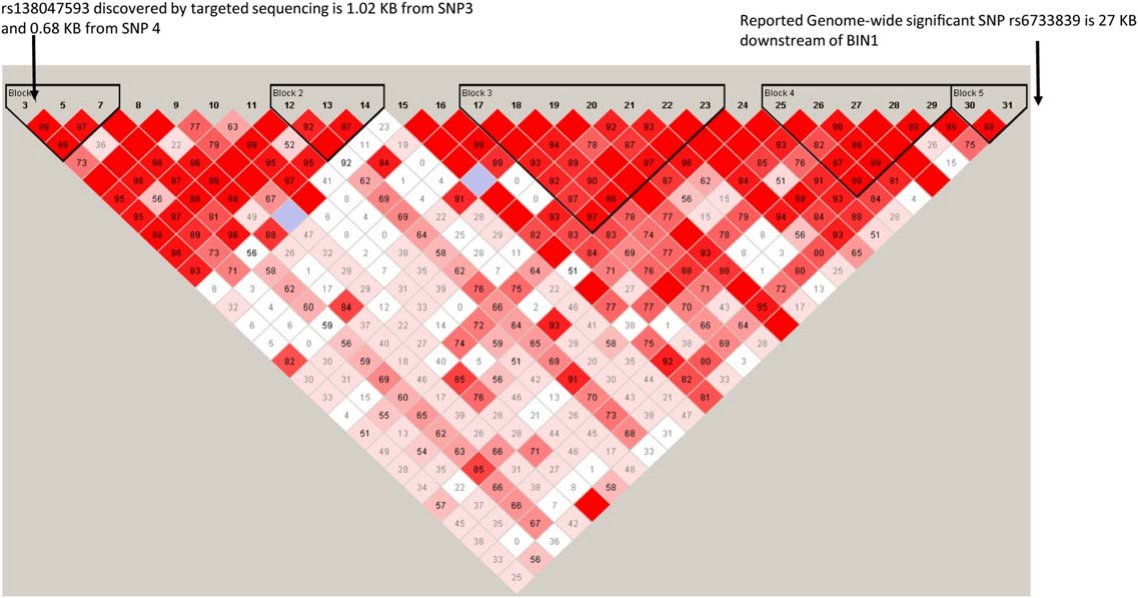
Linkage disequilibrium (LD) plot of *BIN1* in Hispanics. The LD plot is generated using 32 genotyped single nucleotide polymorphisms (SNPs) in 1,675 elderly subjects of Caribbean Hispanic ancestry. The reported genome‐wide significant hit in Lambert et al (rs6733839) is 27.1 kilobases (KB) upstream of *BIN1*.

*BIN1* was also strongly associated in the burden analysis, with damaging variants in 17 cases (5.1%) but no controls. Several SNPs upstream of the *BIN1* locus have been identified in different GWAS, with the largest effect sizes after *APOE* (eg, rs6733839, with a population attributable fraction of 8.1%^35^). *BIN1* transcript levels were increased among LOAD brains compared to controls,^36^ but coding mutations have not been widely explored. So far, there are only 4 *BIN1* coding variants with clinical significance listed in the ClinVar database (p.K575*, p.R154Q, p. D151N, and p.K35N) and all were reported under autosomal recessive centronuclear myopathy. Recently, Tan et al reported that a novel *BIN1* missense mutation p.P318L among the Han Chinese could increase risk of developing AD,^37^ which was not detected in our data sets. The *BIN1* mutations reported here included p.K358R, identified in 8 Caucasian and 6 Hispanic LOAD patients, as well as p.S267L and p.S202T, each identified in a single LOAD patient. None of these mutations were found in controls or unaffected family members. We observed a strong association between LOAD and *BIN1* p.K358R only in the Caribbean Hispanics. The allele frequency of this variant in the Caucasian patients was similar to the general population. *BIN1* p.K358R is a good candidate for functional studies based on its relatively high frequency in familial LOAD cases and segregation in Caribbean Hispanic families. Importantly, *BIN1* p.K358R likely contributes to LOAD independently from the GWAS SNPs, because it is mapped to a different linkage disequilibrium block (Fig 2).

We also identified 6 nonsynonymous variants in *EPHA1*, including p.H888Y, p.R791H, p.V514I, p.R471Q, p.P460L, and p.R337Q. The damaging *EPHA1* variant p.P460L (rs202178565) was identified in cases in all 3 data sets and nearly absent among our controls as well as in 1000 Genomes and the ExAC server data set. This variant segregated with the LOAD in a Caribbean Hispanic family from the Dominican Republic (see Fig 1), supporting its causative role. The *EPHA1* p.P460 amino acid is highly conserved in all mammals and predicted to have a damaging effect on the protein by POLYPHEN estimation. However, the biological impact of this mutation remains to be investigated, because there is only limited information on the function of the protein. Ephrin receptor A1 encoded by *EPHA1* belongs to the ephrin receptor subfamily of the protein–tyrosine kinase family and plays roles in cell and axonal guidance and synaptic plasticity.

A rare variant was found in *MS4A6A*, which affects splicing of 1 transcript of the gene (NM_152852: exon8: c.651+1G>A) and is a missense mutation in another transcript (NM_022349: exon6: c.G652A: p.V218M). The *MS4A6A* p.V218M variant was detected in a single unaffected Caucasian. *MS4A6A* is located among several genes at Chr11q12 that all are associated with the inflammatory response. *MS4A6E* mRNA expression and an SNP nearby the gene (rs670139) are associated with more advanced Braak stages of tangle and plaques in AD brain tissue.^28^ However, until now a functional variant in this region has not been identified, and the current study might provide the first clue.^38^

We identified other rare damaging variants among LOAD associated genes, including *CD2AP* (p.I104N, p.R403G, p.L487V, p.M496I, p.S623N, and p.K633R) and *CLU* (p.V434M). *CD2AP* is an adaptor molecule involved in dynamic actin remodeling and membrane trafficking, and *CLU* encodes clusterin, which is a molecular chaperone,^39^ is present in senile plaques, and has been shown to modulate Aβ oligomer assembly.^40^ We previously reported rare SNPs and small structural variants within the *CLU* gene that were associated with LOAD.^41^

Taken together, the results here imply that multiple rare coding mutations are present in genes identified as LOAD‐associated GWAS loci. Common variants identified in GWAS frequently occur in noncoding sequences within or between genes, and as a result, their functional relationship to disease risk is often hard to define. The data reported here reveal that GWAS loci could harbor both rare damaging variants and common noncoding variants that are independently associated with LOAD (eg, in *CLU*).^41^ Thus, targeted sequencing within GWAS loci may enable the discovery of coding variants underlying or contributing to the association with LOAD. The use of noncoding variants to build cellular and animal models of disease is confounded by uncertainties surrounding the temporally and cell type–specific effects of these noncoding variants on the regulation of gene expression. By contrast, disease‐associated coding sequence variants can be used to build facile, tractable cellular and animal models by a variety of simple methods including both standard transgenesis and clustered, regularly interspaced, short palindromic repeat (CRISPR)‐CAS–based methods. Such models can be used to investigate the underlying molecular mechanisms of these genes in the pathogenesis of LOAD.

The individual effect of these rare variants is expected to be small, and different variants are likely to be causal in different patients and families. For example, the p.K538R variant in *BIN1* has a strong effect in the Hispanic families but was not associated with LOAD in the Caucasian cohort. It is likely that such variants confer modified risk of disease or depend on other interacting genes or environmental factors. Identification of such rare coding variants could thus aid in understanding the biology of the disease.

The strengths of this study are the 3 independent cohorts and the careful phenotyping. That some of the same mutations were observed in 2 or 3 of the cohorts adds validity to our observations. Although there appears to be increased expression associated with some of the genes containing mutations, further studies are required to examine mutation‐specific expression and to understand the mechanisms by which these mutations lead to disease.

## Authorship

B.N.V. and M.G. contributed equally to the article. Senior authors P.S.G.‐H. and R.M. contributed equally to the article. Conception and design of the study: R.M., P.S.G.‐H., and E.R. Data collection: R.M., P.S.G.‐H, E.R., R.L., D.R.‐D., M.M., and I.Z.J.‐V. Data analysis: B.N.V., M.G., C.S., A.K., S.S., and R.C. Writing of the final manuscript: B.N.V., R.M., M.G., P.S.G.‐H, E.R, S.B., J.H.L., and C.R.

## Potential Conflicts of Interest

Nothing to report.

## Supporting information

Additional Supporting Information can be found in the online version of this article.

Supplementary Information
