# Supplementary material for: Rare coding mutations identified by sequencing of Alzheimer disease genome‐wide association studies loci
Source: Ann Neurol. 2015 Jul 28;78(3):487–98. doi: 10.1002/ana.24466 (PMC4546546; doi:10.1002/ana.24466)
Supplement: Supplementary file 1 — Supplementary Information [file ANA-78-487-s001.docx]

**SUPPLEMENTARY TABLE 1: RARE OR NOVEL CODING MUTATIONS FOUND IN LOAD CASES PRIVATE TO EACH DATASET**

**NS-non-synonymous SNV; SG-SNV: stop-gain SNV
These mutations are nominally significant in the Fisher's Exact test when compared with the ExAC data

|  | | | | | | | | | |  |  |
| --- | --- | --- | --- | --- | --- | --- | --- | --- | --- | --- | --- |
| **CHR** | **POS** | **DATASET** | **ID** | **REF** | **ALT** | **# LOAD CASES** | **GENE** | **EXONIC FUNCTION** | **AAChange** | **ExAC_FREQ** | **Fisher.ExAC.P** |
| 19 | 1041922 | HISP | rs146597357 | C | A | 2 | *ABCA7* | NS | NM_019112:c.C253A:p.L85M | 1.02E-04 | 3.49E-03 |
| 19 | 1058263 | HISP | rs142462701 | G | A | 1 | *ABCA7* | NS | NM_019112:c.G5144A:p.R1715H | 0 | 2.96E-02 |
| 19 | 1046287 | HISP | rs150971192 | G | A | 1 | *ABCA7* | NS | NM_019112:c.G1504A:p.G502S | 0 | 2.97E-02 |
| 19 | 1055920 | HISP | rs149196228 | A | G | 1 | *ABCA7* | NS | NM_019112:c.A4220G:p.K1407R | 0 | 2.97E-02 |
| 19 | 1058859 | HISP | rs145071620 | G | A | 1 | *ABCA7* | NS | NM_019112:c.G5320A:p.E1774K | 0 | 2.97E-02 |
| 19 | 1058877 | HISP | rs148891749 | C | T | 1 | *ABCA7* | NS | NM_019112:c.C5338T:p.R1780W | 0 | 2.97E-02 |
| 19 | 1049372 | HISP | . | G | C | 1 | *ABCA7* | NS | NM_019112:c.G2488C:p.D830H | 0 | 2.98E-02 |
| 8 | 27457432 | HISP | rs145083142 | C | G | 1 | *CLU* | NS | NM_001831:c.G1029C:p.E343D | 0 | 2.96E-02 |
| 8 | 27457414 | HISP | . | C | A | 1 | *CLU* | NS | NM_001831:c.G1047T:p.Q349H | 0 | 2.96E-02 |
| 8 | 27461907 | HISP | . | C | T | 1 | *CLU* | NS | NM_001831:c.G835A:p.D279N | 0 | 2.97E-02 |
| 7 | 143091417 | HISP | rs139711610 | C | T | 3 | *EPHA1* | NS | NM_005232:c.G2372A:p.R791H | 0 | 2.55E-05 |
| 7 | 143088789 | HISP | rs138715519 | G | A | 1 | *EPHA1* | NS | NM_005232:c.C2776T:p.R926C | 0 | 2.96E-02 |
| 19 | 1056069 | NIA-LOAD | . | G | A | 2 | *ABCA7* | NS | NM_019112:c.G4243A:p.G1415R | 3.18E-05 | 7.74E-05 |
| 19 | 1055344 | NIA-LOAD | rs145321888 | G | A | 1 | *ABCA7* | NS | NM_019112:c.G4199A:p.R1400H | 2.19E-05 | 9.93E-03 |
| 19 | 1056377 | NIA-LOAD | . | C | T | 1 | *ABCA7* | SG | NM_019112:c.C4465T:p.R1489X | 6.07E-05 | 1.71E-02 |
| 19 | 1047004 | NIA-LOAD | . | T | G | 1 | *ABCA7* | NS | NM_019112:c.T1826G:p.L609R | 9.30E-05 | 3.06E-02 |
| 19 | 1047003 | NIA-LOAD | . | C | G | 1 | *ABCA7* | NS | NM_019112:c.C1825G:p.L609V | 9.31E-05 | 3.06E-02 |
| 19 | 1059059 | NIA-LOAD | rs200308069 | T | G | 1 | *ABCA7* | NS | NM_019112:c.T5438G:p.L1813W | 1.25E-04 | 3.12E-02 |
| 19 | 1061865 | NIA-LOAD | . | G | A | 1 | *ABCA7* | NS | NM_019112:c.G5548A:p.E1850K | 1.24E-04 | 3.15E-02 |
| 19 | 1055101 | NIA-LOAD | . | C | T | 1 | *ABCA7* | NS | NM_019112:c.C3956T:p.S1319L | 1.54E-04 | 3.73E-02 |
| 19 | 1053424 | NIA-LOAD | . | C | T | 1 | *ABCA7* | NS | NM_019112:c.C3317T:p.T1106M | 1.84E-04 | 4.53E-02 |
| 19 | 1046901 | NIA-LOAD | . | A | T | 1 | *ABCA7* | NS | NM_019112:c.A1723T:p.T575S | 1.05E-04 | 4.56E-02 |
| 2 | 127816696 | NIA-LOAD | . | G | A | 1 | *BIN1* | NS | NM_139347:c.C800T:p.S267L | 9.12E-05 | 3.00E-02 |
| 6 | 47501483 | NIA-LOAD | rs149246024 | T | A | 1 | *CD2AP* | NS | NM_012120:c.T311A:p.I104N | 6.13E-05 | 1.70E-02 |
| 8 | 27456017 | NIA-LOAD | . | C | T | 1 | *CLU* | NS | NM_001831:c.G1300A:p.V434M | 6.00E-05 | 1.66E-02 |
| 1 | 207782839 | NIA-LOAD | . | G | A | 1 | *CR1* | NS | NM_000573:c.G4751A:p.R1584Q | 1.05E-04 | 2.66E-02 |
| 7 | 143090798 | NIA-LOAD | . | G | A | 1 | *EPHA1* | NS | NM_005232:c.C2662T:p.H888Y | 1.50E-05 | 6.76E-03 |
| 19 | 1058899 | Toronto |  | C | T | 1 | *ABCA7* | NS | NM_019112:c.C5360T:p.A1787V | 0 | 6.50E-03 |
| 19 | 1054314 | Toronto |  | C | T | 1 | *ABCA7* | NS | NM_019112:c.C3700T:p.R1234C | 0 | 7.04E-03 |
| 19 | 1051531 | Toronto |  | G | A | 1 | *ABCA7* | NS | NM_019112:c.G2908A:p.V970M | 1.54E-05 | 1.28E-02 |
| 19 | 1048950 | Toronto | rs149949633 | G | A | 1 | *ABCA7* | NS | NM_019112:c.G2326A:p.G776R | 1.96E-05 | 1.62E-02 |
| 6 | 47563695 | Toronto | rs200798500 | A | G | 1 | *CD2AP* | NS | NM_012120:c.A1207G:p.R403G | 5.99E-05 | 3.09E-02 |
| 7 | 143096020 | Toronto | rs201581948 | C | T | 2 | *EPHA1* | NS | NM_005232:c.G1010A:p.R337Q | 5.25E-04 | 2.34E-02 |
| 7 | 143095088 | Toronto |  | C | T | 1 | *EPHA1* | NS | NM_005232:c.G1540A:p.V514I | 1.05E-04 | 4.90E-02 |
| 11 | 60268579 | Toronto |  | C | A | 1 | *MS4A12* | NS | NM_017716:c.C338A:p.S113Y | 7.50E-05 | 3.70E-02 |
| 11 | 60296891 | Toronto |  | T | G | 1 | *MS4A13* | NS | NM_001100909:c.T240G:p.F80L | 3.00E-05 | 1.87E-02 |
| 11 | 60165391 | Toronto |  | G | C | 1 | *MS4A14* | NS | NM_001079692:c.G205C:p.G69R | 3.00E-05 | 1.87E-02 |
| 11 | 60160175 | Toronto |  | G | A | 1 | *MS4A7* | NS | NM_021201:c.G564A:p.M188I | 3.00E-05 | 1.87E-02 |
